# Almost half of the RTX domain is dispensable for complement receptor 3 binding and cell-invasive activity of the *Bordetella* adenylate cyclase toxin

**DOI:** 10.1016/j.jbc.2021.100833

**Published:** 2021-05-26

**Authors:** Carlos Angel Espinosa-Vinals, Jiri Masin, Jana Holubova, Ondrej Stanek, David Jurnecka, Radim Osicka, Peter Sebo, Ladislav Bumba

**Affiliations:** 1 Institute of Microbiology, Academy of Sciences of the Czech Republic, Prague, Czech Republic; 2 University of Chemistry and Technology, Prague, Prague, Czech Republic

**Keywords:** adenylate cyclase toxin, *Bordetella pertussis*, CD11b/CD18 integrin receptor, RTX toxin, ACT, adenylate cyclase toxin, CD, circular dichroism, CR3, complement receptor 3, LB, Luria–Bertani, MFI, mean fluorescence intensity, PBS, phosphate buffer saline, RTX, repeat-in-toxin, SAXS, small-angle X-ray scattering

## Abstract

The whooping cough agent *Bordetella pertussis* secretes an adenylate cyclase toxin (CyaA) that through its large carboxy-proximal Repeat-in-ToXin (RTX) domain binds the complement receptor 3 (CR3). The RTX domain consists of five blocks (I–V) of characteristic glycine and aspartate-rich nonapeptides that fold into five Ca^2+^-loaded parallel β-rolls. Previous work indicated that the CR3-binding structure comprises the interface of β-rolls II and III. To test if further portions of the RTX domain contribute to CR3 binding, we generated a construct with the RTX block II/III interface (CyaA residues 1132–1294) linked directly to the C-terminal block V fragment bearing the folding scaffold (CyaA residues 1562–1681). Despite deletion of 267 internal residues of the RTX domain, the Ca^2+^-driven folding of the hybrid block III/V β-roll still supported formation of the CR3-binding structure at the interface of β-rolls II and III. Moreover, upon stabilization by N- and C-terminal flanking segments, the block III/V hybrid-comprising constructs competed with CyaA for CR3 binding and induced formation of CyaA toxin-neutralizing antibodies in mice. Finally, a truncated CyaAΔ_1295-1561_ toxin bound and penetrated erythrocytes and CR3-expressing cells, showing that the deleted portions of RTX blocks III, IV, and V (residues 1295–1561) were dispensable for CR3 binding and for toxin translocation across the target cell membrane. This suggests that almost a half of the RTX domain of CyaA is not involved in target cell interaction and rather serves the purpose of toxin secretion.

The adenylate cyclase toxin (CyaA, AC-Hly, or ACT, Figure 1) is a key virulence factor of the whooping cough agent *Bordetella pertussis* (1, 2). This 1706-residue-long (177-kDa) bifunctional protein comprises a cell-invasive N-terminal adenylyl cyclase (AC) enzyme domain (∼400 residues) that is fused to a pore-forming Repeat-in-ToXin (RTX) hemolysin (Hly) moiety of ∼1300 residues in length (3). The Hly moiety itself consists of several functional domains and undergoes posttranslational activation by a coexpressed CyaC acyltransferase that links fatty-acyl residues to the ε-amino groups of the internal lysine residues K860 and K983 (4, 5, 6). The activated Hly moiety binds and penetrates the plasma membrane of various host cells and delivers into their cytosol the N-terminal AC enzyme domain (7, 8). The AC is activated by cytosolic calmodulin and catalyzes massive conversion of cytosolic ATP to the key second messenger cAMP (9). Unregulated accumulation of cAMP then hijacks cellular signaling and ablates the bactericidal capacities of host phagocytes involved in innate defense to infection (10, 11). Independently of and in parallel to the translocation of the AC domain, the Hly moiety oligomerizes within cell membrane into small cation-selective pores that permeabilize cells and enable efflux of cellular potassium ions (12, 13, 14, 15). This eventually provokes colloid-osmotic (oncotic) cell lysis, such as hemolysis of erythrocytes (16, 17).

**Figure 1:**
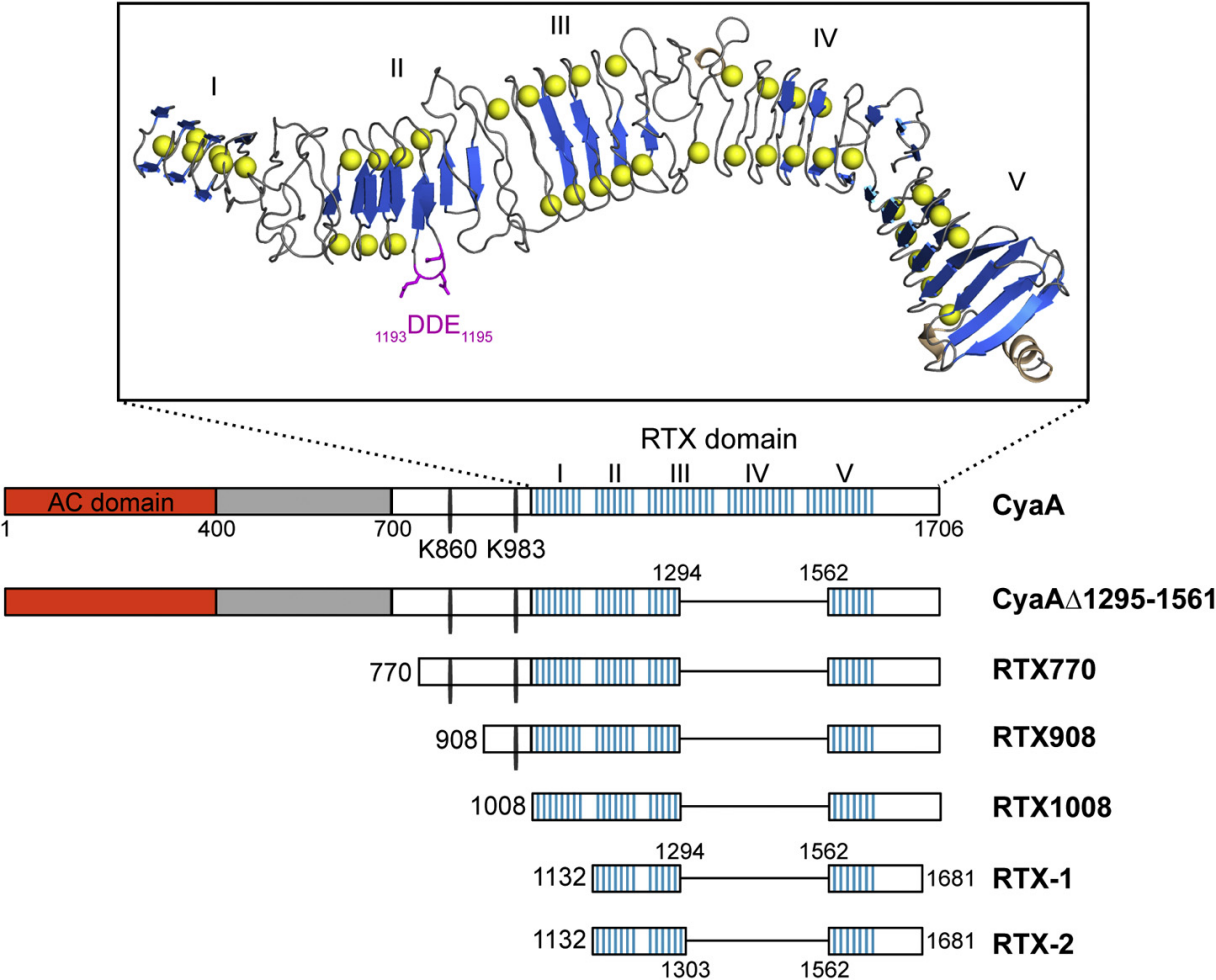
**Schematic representation of the adenylate cyclase toxin (CyaA) and CyaA-derived constructs used in this study.** Intact CyaA consists of the N-terminal enzymatic adenylate cyclase (AC) domain linked to the C-terminal hemolysin (Hly) moiety harboring the hydrophobic pore-forming domain (*gray*), acylation domain with two posttranslationally acylated lysine residues (K860 and K983), and five blocks of calcium-binding RTX repeats with a consensus sequence GGxGxDxxx (*blue vertical lines*). The inset represents a proposed structural model of the RTX domain, comprising the putative CD11b-binding site (*magenta*). The model was previously derived by combining the SAXS data on the full RTX domain and the X-ray structure of the blocks IV and V (24).

The N-terminal part of the Hly moiety harbors the AC-to-Hly linking segment linked to the pore-forming domain followed by the acylated domains (18, 19, 20). These domains jointly form the membrane translocon assembly that mediates the translocation of the AC domain across the lipid bilayer of target cell membrane (21). The second half of the Hly moiety is formed by a large RTX domain that consists of five blocks (I–V) of tandem arrays of characteristic glycine and aspartate-rich nonapeptide (RTX) repeats (22, 23). These bind ∼40 calcium ions with submillimolar affinity and fold highly cooperatively into five RTX β-rolls of antiparallel β-strand assemblies wrapped around the Ca^2+^ ions bound within the turns of the β-rolls (24). The folding of the RTX domain is initiated at a C-terminal scaffold structure and proceeds vectorially from the C-terminal toward the N-terminal end of the protein (25).

CyaA selectively binds the CD11b subunit of the complement receptor 3 (CR3) of phagocytes, also known as the α_M_β_2_ integrin CD11b/CD18, or Mac-1 (26). Toxin binding is initiated by a weak multivalent interaction with the N-linked glycans of CD11b (27, 28, 29), which is followed by a specific recognition of the positively charged loop segment (residues 614–682) of the CD11b subunit by the RTX domain of CyaA (27). Previous work indicated that CR3 recognition by CyaA involves a negatively charged surface (residues 1166–1275) presented at the interface of the folded RTX blocks II and III (30, 31). Deletion analysis then indicated that the structural integrity of the entire RTX domain was required for cell binding and cytotoxic activity of CyaA (21, 32, 33). In particular, the capacity of the RTX domain to undergo the calcium-dependent folding appeared to be critical for the capacity of CyaA to engage the CR3 receptor of phagocytes, penetrate the cellular membrane, and deliver the cytotoxic AC domain into target cell membrane (25). Moreover, acylation of the conserved lysine 983 residue and the structural integrity of the RTX domain of CyaA were found to be crucial also for the capacity of CyaA to trigger production of toxin-neutralizing antibodies in immunized mice (31, 34, 35, 36).

To delimit the structures involved in CR3 binding and protective immunogenicity of the RTX domain, we constructed and characterized a set of RTX fragments that functionally folded in the presence of Ca^2+^ ions and competed with CyaA for CR3 binding. We also show that short and soluble RTX fragments can be used as superior vaccine antigens for induction of CyaA-neutralizing antibodies. The presented results define the CR3-interacting segment of the RTX domain and demonstrate that a 267 residue-large portion of the RTX domain β-rolls is dispensable for CR3 binding and CyaA toxin activities.

## Results

### Ca^2+^-folded RTX domain fragments comprising the interface of β-rolls II and III elicit CyaA-neutralizing antibodies

**Figure 2:**
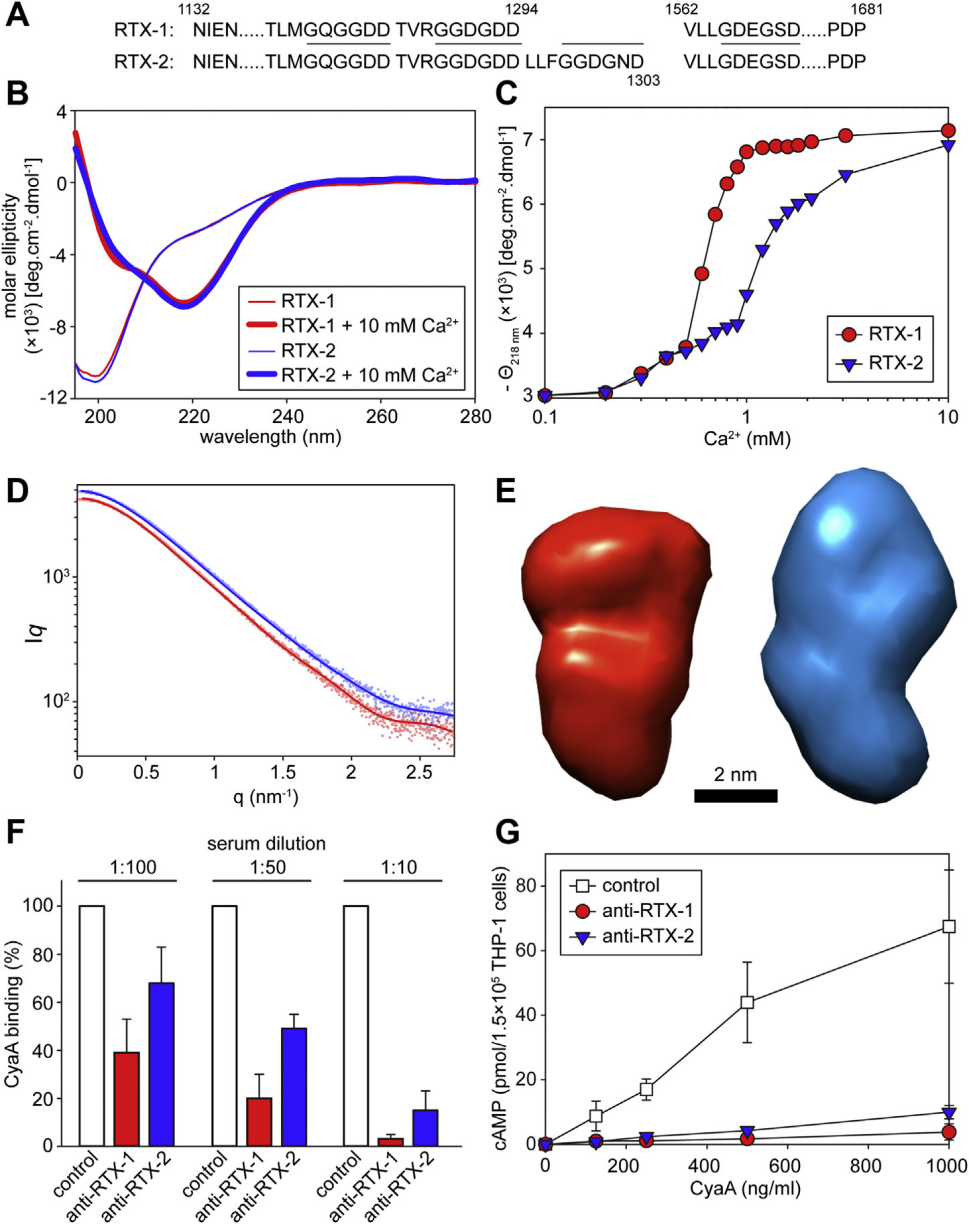
**Structural and functional characterization of the hybrid RTX constructs.***A*, the sequence linkage of the block III and V in the RTX-1 and RTX-2 proteins. The consensus motif GGxGxD is highlighted by the *lines*. *B*, far-UV CD spectra of the RTX-1 (*red*) and RTX-2 (*blue*) proteins in the absence (*thin line*) and the presence (*thick line*) of 10 mM CaCl_2_. *C*, Ca^2+^-induced folding of the RTX-1 (*red dots*) and RTX-2 (*blue triangles*) constructs. The proteins (100 μg/ml) were titrated with CaCl2, and molar ellipticity was followed at 218 nm (Θ218 nm) as a function of Ca^2+^ concentration. *D*, small-angle X-ray scattering data of the Ca^2+^-loaded RTX constructs. Experimental data are shown as *dots*. The theoretical scattering intensities derived from the *ab initio* models are given as continuous *red and blue lines* for RTX-1 and RTX-2, respectively. *E*, the representative SAXS structural models of the Ca^2+^-loaded RTX-1 (*red*) and RTX-2 (*blue*) proteins. *F*, inhibitory activity of sera from mice immunized with the Ca^2+^-loaded RTX-1 (anti-RTX-1) and RTX-2 (anti-RTX-2) proteins. CyaA (1 μg/ml) was preincubated with serum samples at indicated dilutions in DMEM medium for 30 min at 4 °C and the mixture was added to THP-1 cells (10^6^) for additional 30 min at 4 °C. Unbound CyaA molecules were washed and the AC enzyme activity of the cell-bound toxin was determined after the lysis of the cells. CyaA binding was expressed as percentage of toxin binding to THP-1 cells, where 100% corresponds to CyaA binding in the presence of the sera from mock-immunized mice. Data represent the mean ± SD from four independent experiments performed in duplicates. *G*, inhibitory activity of the anti-RTX-1 and anti-RTX2 sera on AC domain translocation. Different concentrations of CyaA (0.31–1000 ng/ml), preincubated in DMEM with serum samples (at 50× dilution) for 30 min at 25 °C, were exposed to THP-1 cells (10^5^/ml) for 30 min at 37 °C and the AC domain translocation of CyaA was assessed by determining the intracellular concentration of cAMP. The data represents the mean ± SD from three independent experiments performed in duplicates.

Previous studies yielded an indirect evidence that the CR3-binding structure of CyaA comprises the folded interface of the RTX β-rolls II and III between residues 1132 and 1355 of CyaA (27, 30, 31). We thus produced the CyaA_1132-1355_ segment as a stand-alone recombinant protein to test its capacity to induce CyaA-neutralizing antibodies, when used as antigen (Fig. 1). However, far-UV circular dichroism (CD) spectroscopic analysis revealed that the CyaA_1132-1355_ polypeptide was unable to undergo the calcium-induced folding and did not form the typical RTX β-roll structures even at >1 mM Ca^2+^ ion concentrations (data not shown). Therefore, we aimed to generate a hybrid RTX block III/V that would fold properly into β-roll due to the presence of the C-terminal folding initiating scaffold structure of block V (25) and could support the folding of the interface linking the RTX β-rolls II and III in CyaA_1132-1355_. In the absence of structural data, it was not possible to reliably predict the resulting orientation of the fused segments of β-rolls III and V, as two consecutive nonapeptide repeats form one turn of the β-roll. Therefore, we engineered two constructs that differed by one nonapeptide repeat at the end of the RTX block III. The two CyaA segments comprising residues 1132–1294 or 1132–1303, respectively, were fused to the C-terminal segment of the block V comprised within residues 1562–1681 of CyaA, yielding the RTX-1 and RTX-2 proteins (Figs. 1 and 2*A*).

Far-UV CD spectra of the RTX-1 and RTX-2 proteins revealed that both polypeptides undergo a Ca^2+^-dependent structural transition from natively disordered conformations into compact β-roll protein structures characterized by prominent negative peaks at 218 nm in the spectra (Fig. 2*B*). However, different Ca^2+^ concentrations were needed for completion of β-roll formation in the two proteins. Folding of RTX-1 was initiated already at ∼0.3 mM Ca^2+^ and was complete at ∼1 mM Ca^2+^. In contrast, completion of RTX-2 folding occurred only at ∼10 mM Ca^2+^ (Fig. 2*C*). This indicated that perturbed structural consecutiveness of the nonapeptide repeats affected the threading and cooperative folding of the consecutive RTX repeats in the RTX-2 construct. In contrast, the characteristic Ca^2+^ loading-driven cooperativeness of vectorial folding was well preserved in the RTX-1 construct.

To corroborate this observation, we determined the low-resolution structures of the Ca^2+^-loaded RTX-1 and RTX-2 proteins in solution using small-angle X-ray scattering (SAXS). The overall structural parameters were calculated from experimental scattering curves (Fig. 2*D*), and the values are displayed in Table 1. The distance distribution functions (*p*_*r*_), derived from scattering intensities (I_*q*_), revealed that the RTX-1 and RTX-2 proteins formed compact objects with asymmetric shapes. The maximum size of the particle derived from the pair-distance distribution function (D_max_) was 7.8 nm for RTX-1 and d 8.0 nm for RTX-2, respectively. The molecular masses of the proteins estimated from the Porod volumes were proportional to those calculated from amino acid sequences and corresponded to monomeric protein species. The low-resolution SAXS models of RTX-1 and RTX-2 revealed elongated protein structures, differing slightly in their shapes (Fig. 2*E*).

**Table 1:** Overall parameters of SAXS data

Sasbdb accession code	RTX-1	RTX-2
	SASDL62	SASDL72
Data collection parameters		
Instrument	P12 (PETRA III)	
Beam geometry (mm^2^)	0.2 × 0.12	
Wavelength (Å)	1.24	
*q* range (nm^−1^)	0.03–4.49	
Concentration range (mg ml^−1^)	1.0–8.0	0.625–5.0
Temperature (K)	283	
Structural parameters		
*I*_0_ [from *P*_*r*_]	4867	3775
*R*_*g*_ (nm) [from *P*_*r*_]	2.33	2.27
*I*_0_ [from Guinier]	4951	3814
*R*_*g*_ (nm) [from Guinier]	2.46	2.34
*D*_max_ (nm)	7.8	8.0
Porod volume estimate (nm^3^)	41	36
Molecular mass determination		
Partial specific volume (cm^3^ g^−1^)	0.724	0.724
Contrast (Δρ × 10^10^ cm^−2^)	3.047	3.047
Molecular mass (kDa) [from *I*_0_]	29	27
Molecular mass (kDa) [from Porod volume]	41	36
Calculated molecular mass from the sequence	33	34
Modeling parameters		
Symmetry	P1	P1
Number of models averaged	20	20
χ^2^	1.37	1.20
Software employed		
Primary data reduction	PRIMUS (40)	
Data processing	GNOM (41)	
*Ab initio* analysis	DAMMIF (42)	
Validation and averaging	DAMCLUST (43)	

To examine whether the interface of RTX β-rolls II/III was properly folded and displayed on its surface, the conformational epitopes that elicit formation of CyaA-neutralizing antibodies, the Ca^2+^-loaded RTX-1 and RTX-2 proteins were used to vaccinate mice. To avoid the Ca^2+^-chelating aluminum hydroxide adjuvant and preserve the Ca^2+^-dependent fold, the proteins were emulsified in Freund’s incomplete adjuvant and 30 μg of the antigens was intraperitoneally injected into mice twice in 2 weeks interval. Mouse immune sera were collected 3 weeks after the second immunization and as shown in Figure 2*F*, by difference to nonimmune control serum, the sera raised against the RTX-1 and RTX-2 proteins blocked CyaA binding to the CR3-expressing human THP-1 monocytic cells in a concentration-dependent manner. The anti-RTX-1 serum was more potent in blocking CyaA binding than the anti-RTX-2 serum. This translated into a higher capacity of the 1:50 diluted anti-RTX-1 serum to inhibit the penetration of CyaA into THP-1 cells and elevate cytosolic cAMP concentration (Fig. 2*G*).

**Figure 3:**
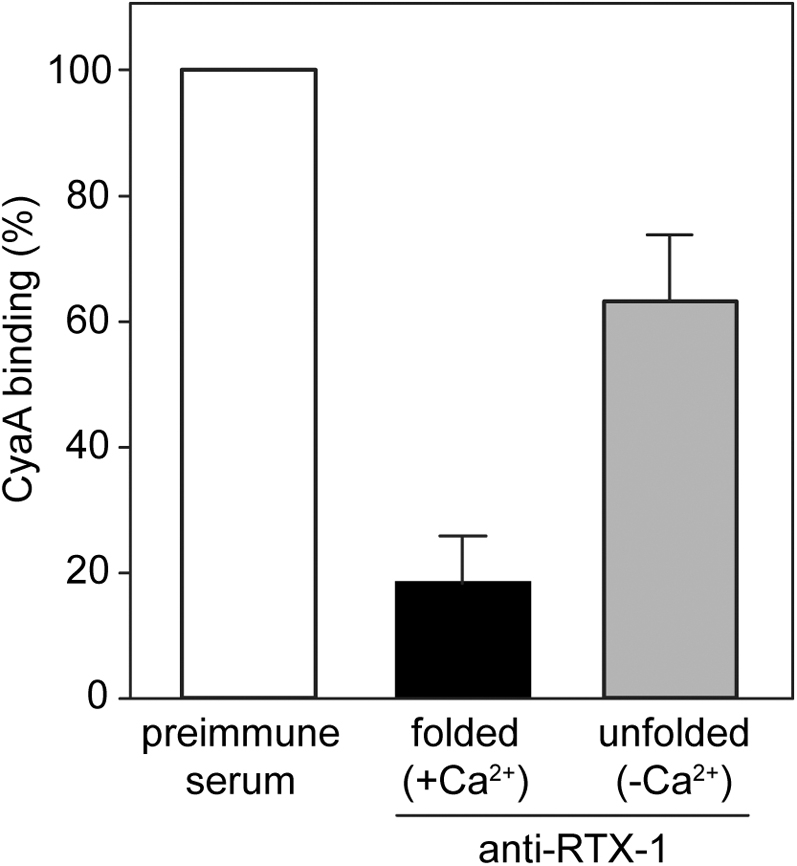
**CyaA-neutralizing antibodies recognize conformation-dependent epitope(s).** Inhibitory activity of sera generated in mice immunized with the unfolded (-Ca^2+^) and the Ca^2+^-loaded (+Ca^2+^) RTX-1 formulated in the presence of Alum and Freund’s incomplete adjuvant, respectively. CyaA binding to THP-1 cells in the presence of 1:50 diluted sera was determined as described in the legend to Figure 2*F*. Data represent the mean ± SD from four independent experiments performed in duplicates.

To assess its vaccine antigen potential, the nonfolded RTX-1 protein was next used for immunization of mice following adsorption to the aluminum hydroxide (alum) adjuvant approved for use in pediatric vaccines. As shown in Figure 1, Figure 3, 1:50 diluted sera from mice immunized with alum-adjuvanted RTX-1 (-Ca^2+^) were less potent in inhibition of CyaA binding to THP-1 cells than the 1:50 diluted sera raised against the Ca^2+^-loaded RTX-1 in Freund’s adjuvant (Ca^2+^). This difference could be due to the different adjuvant potencies and immune response shaping effects elicited by the two different adjuvants. Alternatively, this result could also indicata a limited propensity of the RTX-1 protein to fold into a stable and immunogenic β-roll structure upon release from alum into the body fluids containing the physiological 2 mM Ca^2+^ concentrations.

### N-terminal extensions increase the stability of the of RTX-1 protein scaffold

To enhance the folding capacity and structural stability of the RTX-1 antigen, we extended its termini by addition of portions of the CyaA polypeptide. Three additional constructs of increasing length were generated by (i) extending the N-terminal portion of RTX-1 up to CyaA residue 1008 through addition of residues of RTX block I (RTX1008); (ii) up to residue 908 by further adding a fragment of the acylated domain of CyaA (RTX908); or (iii) by extending the protein up to residue 770 by addition of the entire acylated domain of CyaA (RTX770). All constructs were also extended on the C-terminal end up to the last residue of CyaA through addition of the secretion signal segment (*c.f.* Fig. 1). All the proteins were produced in *E. coli* cells in the presence of the CyaC acyltransferase that catalyzes the posttranslational fatty-acylation of ε-amino groups of the Lys860 and Lys983 residues (5) and purified close to homogeneity by anion-exchange chromatography from urea extracts of the inclusion bodies (37). The acylation status of the proteins was then characterized by mass spectrometry, which revealed that both Lys860 and Lys983 residues of RTX770 and the Lys983 of the RTX908 were predominantly modified by a mixture of palmitoyl (C16:0) and palmitoleyl (C16:1) chains, with a small proportion of myristoyl (C14:0) and octadecenoyl (C18:1) groups (data not shown). Hence, the overall acylation status of the acylated proteins resembled that of intact recombinant CyaA (6).

**Figure 4:**
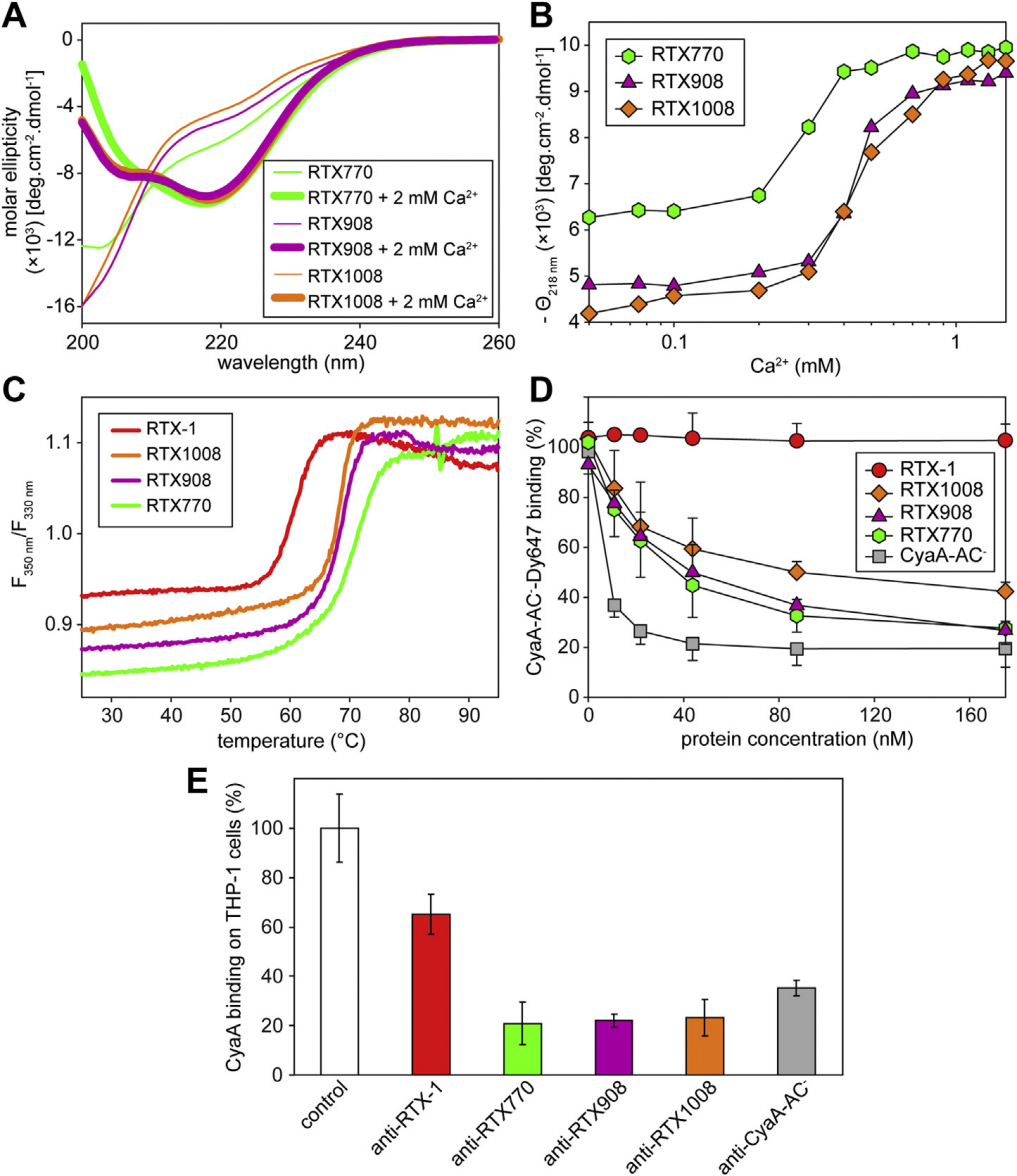
**Acylation domain of CyaA provides the stability and antigenicity of the hybrid RTX-1 construct.***A*, Far-UV CD spectra of the RTX770 (*green*), RTX908 (*violet*), and RTX1008 (*orange*) proteins in the absence (*thin line*) and the presence of 10 mM CaCl_2_ (*thick line*). *B*, Ca^2+^-induced folding of RTX770 (*green circles*), RTX908 (*violet triangles*), and RTX1008 (*orange squares*). The proteins (100 μg/ml) were titrated with CaCl_2_, and molar ellipticity was followed at 218 nm (Θ218 nm) as a function of Ca^2+^ concentration. *C*, thermal unfolding of Ca^2+^-loaded RTX-1 (*red*), RTX770 (*green*), RTX908 (*violet*), and RTX1008 (*orange*) as assessed by nanoDSF. The ratios of fluorescence intensities at 350 nm and 330 nm (F_350_/F_330_) after heating of the protein samples (200 μg/ml) were followed as function of temperature. *D*, competitive binding of RTX-1 (*red*), RTX770 (*green*), RTX908 (*violet*), and RTX1008 (*orange*) to the CHO-CR3 cells. The CHO-CR3 cells, preincubated with the indicated concentrations of the different proteins for 30 min at 4 °C, were stained with Dy647-labeled CyaA-AC^−^ (5.6 nM) and the fluorescence intensity of cell-associated CyaA-AC^−^ was analyzed by flow cytometry. The data are expressed as % of Dy647-labeled CyaA-AC^−^ binding, where 100% corresponded to CyaA-AC^−^ binding in the absence of the competitor. Data represent the mean ± SD from three independent experiments. *E*, inhibitory activity of sera from mice immunized with Alum-adjuvanted, Ca^2+^-free RTX-1 (anti-RTX-1), RTX770 (anti-RTX770), RTX908 (anti-RTX908), RTX1008 (anti-RTX1008), and CyaA-AC (anti-CyaA-AC^−^) proteins. CyaA binding to THP-1 cells in the presence of 1:50 diluted sera was determined as described in the legend to Figure 2*F*. Data represent the mean ± SD from four independent experiments performed in duplicates.

The far-UV CD spectra revealed that all three proteins underwent the characteristic Ca^2+^-induced structural transition from an unfolded into a compact protein structure (Fig. 4*A*). However, compared with the spectra of the RTX908 and RTX1008 proteins, the Ca^2+^-free RTX770 protein exhibited a less intense negative band at 200 nm and a more pronounced negative shoulder at 222 nm. This indicated partial folding of the N-terminal non-RTX portion of the RTX770 construct that can be predicted to adopt an α-helical structure and would thus fold independently of Ca^2+^ ions. Moreover, the apparent affinity of the RTX770 protein for Ca^2+^ ions appeared to be higher than that of the RTX908 and RTX1008 proteins, suggesting that folding of the N-terminal segment of RTX770 stabilized the fold of the C-terminal β-roll structure (Fig. 4*B*).

The overall stability of the individual Ca^2+^-loaded construct folds was examined by thermal unfolding experiments using nanodifferential scanning fluorimetry (nanoDSF). The midpoints of the thermal melting curves (T_*m*_) of RTX-1 and RTX1008 differed by 8 °C, with unfolding of RTX-1 occurring with a T_*m*_ of 60 °C compared with the higher T_*m*_ of 68 °C determined for RTX1008 (Fig. 4*C*). The longer and partially acylated RTX908 protein exhibited a similar T_*m*_ value of about 69 °C, while the even longer RTX770 construct unfolded at an increased temperature with a T_*m*_ value of 72 °C. The above data showed that RTX1008, having the RTX β-rolls I and II intact, was more stable than RTX-1, lacking the β-roll I. Hence, additions of an N-terminal portion comprising the K983 acylation site in RTX908, or the entire acylation domain, bearing both acylated K860 and K983 lysine residues in RTX770, substantially increased the stability of the folded Ca^2+^-loaded proteins.

Therefore, we assessed the capacity of the RTX constructs to outcompete the fluorescently (Dy647)-labeled CyaA-AC^−^ from binding to the CR3 receptor. In contrast to unlabeled CyaA-AC^−^ toxoid, which efficiently outcompeted the Dy647-labeled CyaA-AC^−^ from CR3 in a dose-dependent manner, the RTX-1 protein did not inhibit binding of the labeled toxoid to CHO-CR3 cells. The RTX770 and RTX908 proteins were then more potent in CR3 binding and competitive blocking of Dy647-labeled CyaA-AC^−^ binding than was RTX1008, although all three constructs competed less effectively than the unlabeled full-length CyaA-AC^−^ toxoid (Fig. 4*D*). These results showed that the truncated RTX constructs specifically bound the CR3 receptor. Hence, the deleted portion of the RTX block III, the entire block IV, and the N-terminal portion of the block V (residues 1295–1561), missing in the RTX1008, RTX908, and RTX770 proteins, were not required for CR3 binding.

To assess whether the above described RTX constructs could serve as antigens for induction of CyaA-neutralizing antibodies, the nonfolded proteins were admixed with alum adjuvant and used as vaccines. Groups of mice were immunized with the antigens and the CyaA-neutralizing activity of the collected sera was assessed. As shown in Figure 1, Figure 4*E*, 1:50 diluted sera of mice immunized with the RTX1008, RTX908, and RTX770 proteins, respectively, reduced the CyaA binding to THP-1 cells by 80%. The 1:50 diluted anti-RTX-1 sera caused only ∼35% reduction of CyaA binding, compared with nonimmune control sera. Hence, the enhanced folding propensity of the larger RTX constructs in the Ca^2+^-containing body fluids enabled them to adopt a conformation that displayed the conformational epitopes of the CR3 binding site *in vivo* and induced CyaA-neutralizing antibodies in mice at least as efficiently as the intact CyaA-AC^−^ toxoid applied at equimolar amount as a positive control (*c.f.* Fig. 4*E*).

### Residues 1295–1561 of the Ca^2+^-binding RTX domain are dispensable for cytotoxic activity of CyaA

**Figure 5:**
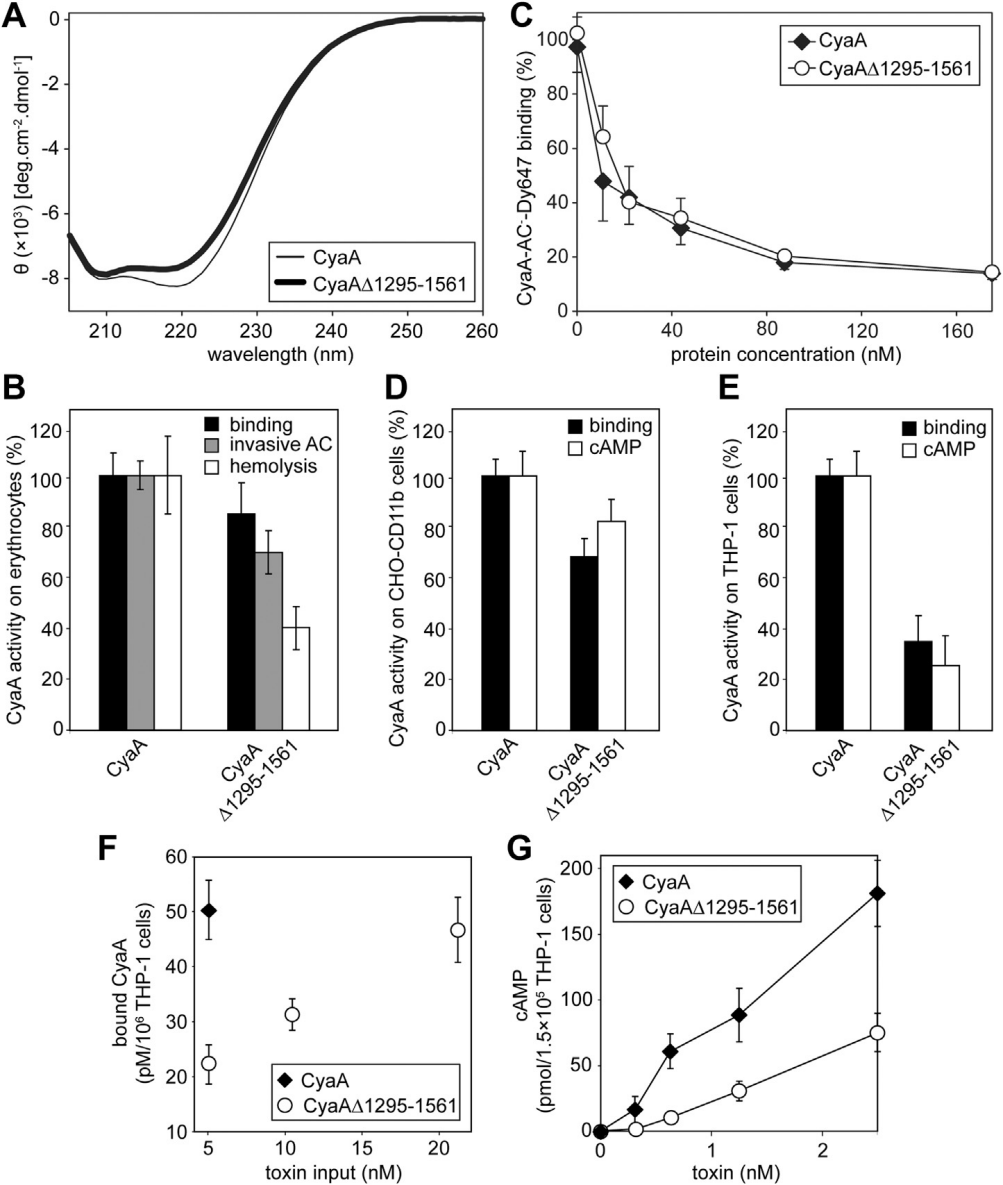
**Residues 1295–1561 of CyaA are dispensable for biologic activity of CyaA.***A*, far-UV CD spectra of the CyaA (*thin line*) and CyaA_Δ1295-1561_ (*thick line*) in the presence of 5 mM CaCl_2_. *B*, biological activity of CyaA_Δ1295-1561_ on erythrocytes. Sheep erythrocytes (5 × 10^8^/ml) were incubated with CyaA proteins (5 nM) in the presence of 2 mM CaCl_2_ at 37 °C for 30 min before the binding and invasive AC activity of the toxins were determined by AC assays. For hemolytic activity, sheep erythrocytes (5 × 10^8^/ml) were incubated with 50 nM of the proteins in the presence of 2 mM CaCl_2_ at 37 °C for 4 h and the release of hemoglobin was determined spectrophotometrically at 541 nm. Data represent the mean ± SD from four independent experiments performed in duplicates. *C*, competitive binding of CyaA_Δ1295-1561_ to CHO-CR3 cells. The cells were incubated with the increasing concentrations of CyaA and CyaA_Δ1295-1561_ for 15 min at 4 °C before CyaA-AC^−^-Dy647 (5.6 nM) was added for additional 30 min and the fluorescence intensity of cell-associated CyaA-AC^−^-Dy647 was analyzed by flow cytometry. The data are expressed as % of CyaA-AC^−^-Dy647 binding, where 100% corresponded to CyaA-AC^−^-Dy647 binding in the absence of the competitor. Data represent the mean ± SD from five independent experiments. *D*, biological activity of CyaA_Δ1295-1561_ on CHO-CR3 cells. CyaA binding was assessed by determining the amount of cell-associated AC enzyme activity after incubation of the CHO-CR3 cells (10^6^/ml) with 5 nM CyaA proteins at 4 °C for 30 min. AC domain translocation (cAMP intoxication) was assessed by determining intracellular concentrations of cAMP generated in CHO-CR3 cells exposed to different CyaA concentrations (0.25, 0.125, 0.062, 0.031, and 0.015 nM) at 37 °C for 30 min. Activities of the proteins are expressed in %, where 100% corresponded to the activity of intact CyaA. The data represent mean ± SD from four independent experiments performed in duplicates. *E*, biological activity of CyaA_Δ1295-1561_ on THP-1 cells. CyaA binding was assessed by determining the amount of cell-associated AC enzyme activity after incubation of the THP-1 cells (10^6^/ml) with 5 nM CyaA proteins at 4 °C for 30 min. AC domain translocation was assessed by determining intracellular concentrations of cAMP generated in THP-1 cells exposed to different CyaA concentrations (1.25, 0.62 and 0.31 nM) at 37 °C for 30 min. Activities of the proteins are expressed in %, where 100% corresponded to the activity of intact CyaA.The data represent mean ± SD from four independent experiments performed in duplicates. *F*, a high dose of CyaA_Δ1295-1561_ can compensate the reduced binding of the protein. THP-1 cells (10^6^/ml) were incubated with indicated concentrations of the CyaA proteins at 4 °C for 30 min and the amount of cell-associated AC enzyme activity was determined. Data represent the mean ± SD from three independent experiments performed in duplicates. *G*, dose-dependent intoxication of THP-1 cells induced by CyaA proteins. The intracellular concentrations of cAMP were determined after incubation of THP-1 cells (1.5 × 10^5^/ml) with indicated concentrations of the CyaA proteins at 37 °C for 30 min. Data represent the mean ± SD from three independent experiments performed in duplicates.

The above results suggested that the segment comprising the residues 1295–1561 of the RTX domain might be dispensable also for CR3 binding of the entire CyaA toxin. Therefore, we constructed and purified a CyaA_Δ1295-1561_ toxin having the 267 residues deleted. As shown in Figure 5*A*, the Ca^2+^-loaded CyaA_Δ1295-1561_ protein exhibited the double-negative peak at 209 and 217 nm in the far-UV CD spectrum, witnessing Ca^2+^-dependent formation of the β-roll structures in the truncated RTX domain of CyaA_Δ1295-1561_. Despite the lower amplitude of the 217-nm peak, reflecting the absence of a segment corresponding to two out of five Ca^2+^-binding β-roll structures of the RTX domain, the CyaA_Δ1295-1561_ protein responded to the presence of Ca^2+^ ions like the intact CyaA. Moreover, the structure of the CyaA_Δ1295-1561_ protein was not grossly affected, since it was capable to bind in a CR3 receptor-independent manner to sheep erythrocytes only slightly less well (∼80%) than intact CyaA (Fig. 5*B*). Indeed, CyaA_Δ1295-1561_ exhibited only a mildly reduced (∼70%) specific invasive AC activity, measured as the capacity of the toxin to translocate the AC enzyme domain across the plasma membrane of erythrocytes into cell cytosol, where the AC enzyme becomes inaccessible to inactivation by externally added trypsin. Furthermore, the CyaA_Δ1295-1561_ protein also formed pores within erythrocyte membrane and provoked hemolysis of erythrocytes, albeit with about twice lower specific hemolytic potency than intact CyaA (Fig. 5*B*).

A somewhat more pronounced decrease of the specific toxin activity of CyaA_Δ1295-1561_ was observed on the CR3-expressing cells in function of the specific level of CR3 expression on the various types of target cells. On transfected CHO cells, which express very high levels of the CR3 receptor, the truncated toxin competed effectively for CR3 binding with CyaA-AC^−^-Dy647 at higher than 10 nM concentrations (Fig. 5*C*). Moreover, the truncated CyaA_Δ1295-1561_ toxin bound and intoxicated the CR3-expressing CHO cells to comparable levels as the wild-type CyaA (Fig. 5*D*). Presumably due to reduced levels of CR3 on cell surface, the binding of CyaA_Δ1295-1561_ to THP-1 cells was less efficient, with ∼40% CyaA_Δ1295-1561_ binding compared with intact CyaA (Fig. 5*E*). The reduced amounts of cell-associated CyaA_Δ1295-1561_ molecules then translated into lower levels of intracellular cAMP accumulation in THP-1 cells (∼20–30% of the cAMP formed by intact CyaA). As indeed shown in Figure 5*F*, about fourfold higher input concentration of CyaA_Δ1295-1561_, than of the intact CyaA, was required for achieving of equal cell-bound amounts of the two toxins on THP-1 cells. Under such conditions, however, the intracellular cAMP levels generated by the AC enzyme delivered by the CyaA_Δ1295-1561_ protein added at 2.5 nM concentration were roughly comparable to that delivered by 0.62 nM intact CyaA (Fig. 5*G*). These results show that the deletion of 267 residues from the RTX domain of CyaA somewhat reduced the affinity of CR3 binding of the CyaA_Δ1295-1561_ protein on THP-1 cells, but at an equal amount of membrane-bound protein, the specific capacities of the mutant and intact toxins to translocate the AC domain across the membrane of target cells were similar.

## Discussion

We exploited the low-resolution SAXS structure-based model of the RTX domain of CyaA (24) for semirational design of small foldable RTX proteins. These lack the residues 1295–1561 of the RTX domain but still retained the capacity to undergo the vectorial Ca^2+^-driven folding that yields formation of RTX β-rolls when the segments of the truncated RTX block III and V were linked in the folding frame to enable the vectorial threading of the RTX repeats into the β-roll wrap around the bound calcium ions. Hence, an engineered hybrid RTX block III/V β-roll formed in the RTX-1 scaffold then supported folding of the neighboring CR3-binding site structure at the interface of β-roll blocks II and III. This was preserved well enough to trigger the formation of toxin-neutralizing antibodies that blocked CyaA binding to the CR3 receptor on the surface of target cells. Moreover, N-terminally extended and more stable constructs derived from the RTX-1 scaffold exhibited a superior folding capacity, enhanced thermal stability, and increased capacity to trigger toxin-neutralizing antibodies in mice. These small and soluble RTX1008, RTX908, and RTX770 proteins thus represent promising antigens for formulation into a next-generation pertussis vaccine that would induce also CyaA toxin-neutralizing antibodies.

The here presented results demonstrate for the first time that a 267 residue-long segment (residues 1295–1561), corresponding to nearly a half of the RTX domain of CyaA and covering portions of the RTX blocks III and V plus the entire block IV, is dispensable for CyaA toxin function. The CyaA_Δ1295-1561_ toxin exhibited a somewhat reduced affinity of binding to the CR3 receptor, but was capable to bind and translocate across erythrocyte membrane. Moreover, CyaA_Δ1295-1561_ was also capable to provoke hemolysis of erythrocytes, thus reflecting its cell-permeabilizing pore-forming activity. The specific hemolytic activity of CyaA_Δ1295-1561_ was reduced compared with intact CyaA due to a somewhat reduced erythrocyte-binding capacity. Indeed, the pore-forming activity depends on oligomerization of the toxin, and it is a higher-order function of the number of membrane-associated CyaA molecules, with a Hill number of 3–4 (35, 38). Hence, even a small difference of the amount of cell-bound toxin yields a notable difference in its pore-forming (cell-permeabilizing) hemolytic activity.

Most importantly, when bound to transfected CHO cells expressing high levels of the CR3 receptor, or upon equalizing the amounts of cell-bound toxin by higher input concentration of the truncated protein, the CyaA_Δ1295-1561_ toxin translocated its AC domain across target cell membrane as efficiently as the intact CyaA (*c.f.* Fig. 5*F*). Moreover, the CyaA_Δ1295-1561_ toxin, and less efficiently (at higher concentrations) also the RTX1008, RTX908, and RTX770 proteins, competed with the CyaA-AC^−^ toxoid for binding to the receptor CR3. Hence, the vectorial folding signal was adequately transmitted in-phase through the engineered block III/V hybrid β-roll structure, from the C-terminal folding-initiating structure of block V throughout the rest of the RTX-1 scaffold (25). Such cooperative threading of the RTX repeats into the β-roll wrap around the bound Ca^2+^ ions in the RTX-1 scaffold then allowed the formation of a properly structured CR3-binding site at the block II/III interface.

In contrast, folding was largely impaired in the RTX-2 scaffold due to the presence of the _1295_LLFGGDGND_1303_ nonapeptide at the junction of the arrays of tandem RTX repeats blocks III and V (Fig. 2). The atomic structures of the RTX-1 and RTX-2 proteins have not been determined, but the distinct behavior of these two constructs could be plausibly ascribed to the difference in “consecutiveness” of the RTX motifs, with the the RTX blocks III and V being linked “out-of-frame” in the hybrid RTX-2 construct. Since the parallel β-roll is built up by the succession of turns, each consisting of two consecutive RTX repeats, a deletion, or insertion of a nonapeptide motif in the turn leads to a “folding frameshift.” This must not necessarily impact the Ca^2+^-induced assembly of a single block of RTX repeats. However, it will definitely affect the folding of the successive block(s) of RTX repeats, as these appear to form a single continuous β-helical structure (*c.f.* Fig. 1) that is connected by nonrepetitive β-strand linker segments (24). Such “folding frameshift” would then lead to twisting of the turns of the β-helix and most likely would result in improper folding and violation of the β-roll structure assembly. Indeed, this appeared to be the case of the RTX-2 construct. It required an increased Ca^2+^ concentrations for initiation of folding, and this was not complete even at 10 mM Ca^2+^ concentration. In line with that, RTX-2 also exhibited a lower capacity to elicit CyaA-neutralizing antibodies than RTX-1 (*c.f.* Fig. 2, *F* and *G*).

Indeed, structural integrity and proper conformation of the RTX repeat domain were previously shown to be essential for the protective antigenicity of CyaA (34, 36). Moreover, Wang *et al*. (31) demonstrated that epitopes targeted by CyaA-neutralizing antibodies in the RTX domain are conformational and that their recognition depends on the Ca^2+^-induced assembly of the RTX β-roll structures. This goes well with our data showing that neutralizing activity of anti-RTX-1 sera was higher when mice were immunized with the folded and Ca^2+^-loaded protein, emulsified in oil, compared with vaccination of mice with nonfolded RTX-1 adsorbed to alum (Fig. 3). Even if the impact of different adjuvants used in the two preparations could not be excluded, additional support for the conclusion on the structural requirements for induction of CyaA-neutralizing antibodies is provided by the results obtained with the longer and more stably folding RTX-1-derived constructs. Thermal denaturation curves revealed that the melting temperature (T_*m*_) of RTX-1 was by 8–9 °C lower than that of the RTX1008 and RTX908 and by 14 °C lower than that of the RTX770 protein. Hence, the N-terminal extension of RTX-1 by the RTX block I (RTX1008), or by a fragment of, or an entire acylated domain (RTX908 and RTX770), substantially increased the stability of the functional fold of the hybrid proteins. In line with that, the sera from mice immunized with equimolar amounts of the RTX1008, RTX908, and RTX770 proteins were at least as potent in toxin neutralization, as the sera of mice vaccinated with the full-length intact CyaA-AC^−^ toxoid. Hence, all three hybrid RTX constructs displayed the epitopes of the receptor-binding site targeted by the antibodies capable to block CyaA binding to CR3.

The CR3-binding site of CyaA was initially mapped between the residues 1166–1281 located at the interface of the RTX block II and III (30). Recently, we proposed that binding of CyaA to the CR3 receptor is mediated by an electrostatic interaction between the negatively charged CyaA residues D1193, D1194, and E1195 and the positively charged residues of the segment between residues 614–682 of the CD11b subunit of the CR3 receptor (27). Indeed, recent template-based homology modeling of the entire RTX domain of CyaA revealed that the negatively charged _1193_DDE_1195_ tripeptide would form an extended loop facing outward from the β-roll structure (24). It would thus be optimally placed for initiation of the intermolecular interaction with the positively charged loop of CD11b. However, an electrostatic interaction would likely not be sufficient for assuring toxin binding to CD11b with sufficient affinity. Additional residues of the receptor-binding interface of CyaA are thus most likely engaged in the interaction as well. Moreover, CyaA appears to exhibit a weak lectin activity and binds the CR3 receptor through a multivalent initial interaction with multiple N-linked glycan chains located in the C-terminal portion of the CD11b subunit (28, 29). On the other hand, the here presented results obtained with the RTX-1-derived constructs lacking a large part of the RTX domain reveal that the specificity of the CyaA-CR3 interaction is mostly likely determined by the residues comprised at the interface of RTX blocks II and III. This segment is, indeed, targeted by the toxin-neutralizing antibodies M1H5 and M2B10 that competitively inhibit CyaA binding to CR3 and bind nonoverlapping epitopes of the RTX block II and III interface (31). Moreover, the residue D1194 involved in CR3 binding is also part of the M1H5 epitope (31).

The importance of the interface of RTX blocks II and III as the principal CR3-binding site on CyaA is further supported by the here reported observation that the deletion of 267 residues from the RTX domain did not affect the capacity of the CyaA_Δ1295-1561_ protein to interact with CR3 any importantly. The moderate reduction of the CyaA_Δ1295-1561_ binding to THP-1 cells would suggest that the missing segment of the RTX domain (residues 1295–1561) might be contributing to the polyvalent low-affinity interactions of the RTX domain with glycan chains attached to the CR3 heterodimer. However, the functional defect of CyaA_Δ1295-1561_ was rather mild, showing that the deleted segment does not play any important role in toxin binding and translocation across the plasma membrane of target cells. This conclusion goes well with our recent results showing that the entire RTX domain of CyaA can be functionally replaced by the much shorter RTX domain of the *E. coli* HlyA (21). Such chimeric CyaA/HlyA molecule was retargeted for binding to the LFA-1 integrin (CD11a/CD18) of leukocytes and was still capable to deliver the AC domain into cytosol of erythrocytes as well as of LFA-1-expressing cells. This indicates that the RTX domain plays no role in membrane translocation of the AC domain but is important for excretion of the toxin out of the bacterial cells and for mediating of binding of the toxin to the surface receptors of specific target cells, thus increasing the probability of membrane insertion and translocation of the toxin into their cytosol.

## Experimental procedures

### Cells and growth conditions

Chinese hamster ovary cells expressing the human CD11b/CD18 (CHO-CR3) (27) and human monocytic THP-1 cell line (ATCC TIB-202) were cultured at 37 °C under a humidified 5% CO_2_ atmosphere in F12 (Gibco Invitrogen, USA) and RPMI 1640 (Sigma, USA) medium supplemented with 10% (v/v) fetal calf serum (GIBCO Invitrogen, USA) and Antibiotic Antimycotic Solution (Sigma, USA), respectively. *E. coli* XL1-Blue (Stratagene) and BL21 λ(DE3) strains transformed with the appropriate vectors were grown in Luria–Bertani (LB) medium or LB agar plates in the presence of kanamycin (60 μg/ml) or ampicillin (150 μg/ml).

### Plasmid constructs

The pET42b-TEV-RTX-1 and pET42b-TEV-RTX-2 constructs, encoding the RTX-1 and RTX-2 proteins fused to a glutathione S-transferase (GST) fusion protein through a tobacco etch virus (TEV) protease cleavage site, were prepared by PCR mutagenesis. The nucleotide sequence of the *cyaA* gene encoding the residues 1132–1294 (for RTX-1) and 1132–1303 (for RTX-2) and 1562–1681 (for both constructs) was amplified by PCR using pairs of primers (5′-TTTCCATGGAAAACCTGTACTTCCAGGGCAATATCGAGAATCTGCACGG-3′ and 5′-TTTGACGTCATCGCCGTCGCCGCCG-3′, and 5′-AAAGACGTCCTGCTGGGCGACGAGG-3′ and 5′-TTTCTCGAGTCAGGGGTCCGGATACTGC-3′ for RTX-1 and 5′-TTTCCATGGAAAACCTGTACTTCCAGGGCAATATCGAGAATCTGCACGG-3′ and 5′-TTTGACGTCGTTGCCGTCGCCGCCG-3′, and 5′-AAAGACGTCCTGCTGGGCGACGAGG-3′ and 5′-TTTCTCGAGTCAGGGGTCCGGATACTGC-3′ for RTX-2, respectively), digested with the respective pair of restriction enzymes (NcoI/AatIII and AatII/XhoI) and ligated to the NcoI/XhoI-cleaved pET42b vector.

The expression vectors encoding the RTX770, RTX908, and RTX1008 proteins were derived from pT7CT7ACT1-ΔNdeI, a bicistronic vector encoding the structural *cyaA* gene and the *cyaC* gene for the dedicated acyltransferase (8). For construction of the pT7CT7-RTX770, pT7CT7-RTX908, and pT7CT7-RTX1008 plasmids, the PCR fragments amplified from pT7CT7ACT1-ΔNdeI using the reverse 5′-CCAGAGCTCGTTGTCCTGG-3′ and the forward 5′-ATACATATGCATCATCATCATCATCATAGCAGCGTGATCGGGGTG-3′ (RTX770), 5′-ATACATATGCATCATCATCATCATCATAAACTGGATGTGATCGGCGG-3′ (RTX908) and 5′-ATACATATGCATCATCATCATCATCATGAGCATGTGCAGCACATCAT-3′ (RTX1008) primers were digested with NdeI/SacI and ligated together with the 782-bp SacI/AccIII fragment of pET42b-TEV-RTX-1 into the NdeI/AccIII-cleaved pT7CT7ACT1-ΔNdeI vector.

The pT7CT7ACT1_Δ1295-1561_ plasmid was prepared by the ligation of the ScaI/SacI fragment of the pT7CT7-RTX1008 plasmid (2007 bps) into the ScaI/SacI-cleaved pT7CT7ACT1-ΔNdeI vector (6095 bps).

All the plasmid constructs were confirmed by DNA sequencing using ABI Prism 3130XL analyzer (Applied Biosystems, USA) using a Big Dye Terminator cycle sequencing kit.

### Production and purification of proteins

The RTX-1 and RTX-2 proteins were expressed as the C-terminal GST fusion proteins in *E. coli* BL21 λ(DE3) cells grown at 37 °C in MDO medium (M9 salts, 20 g/l of yeast extract and 20 g/l of glycerol) supplemented with 60 μg/ml kanamycin after induction of the bacterial cells with 0.5 mM isopropyl β-D-1-thiogalactopyranoside (IPTG) at optical density at 600 nm of ∼0.6. After 4 h, the cells were harvested by centrifugation (4000*g* for 20 min at 4 °C), resuspended in PBS, and disrupted by sonication. The cell lysates were clarified by centrifugation (30,000*g* for 30 min at 4 °C) and loaded on a glutathione agarose column (Life Technologies, USA) equilibrated with phosphate buffer saline (PBS). The column was extensively washed with PBS and the GST fusion proteins were eluted with 10 mM reduced glutathione in PBS. Collected fractions were pooled, mixed with purified recombinant TEV protease (1:20 w/w), and dialyzed at 4 °C overnight against the buffer containing 20 mM Tris-HCl (pH 7.4) and 150 mM NaCl. The protein mixture was incubated at 70 °C for 30 min, and the precipitate was removed by centrifugation at 30,000*g* for 15 min. The supernatant was concentrated by using Amicon YM10 ultrafiltration membrane and loaded on a Superdex 200HR gel filtration column (GE Healthcare, United Kingdom) equilibrated with a buffer containing 10 mM Tris-HCl (pH 7.4) and 150 mM NaCl. Collected fractions were concentrated by ultrafiltration using Amicon YM10 membrane (Millipore, USA) and stored at –20 °C.

The wild-type CyaA toxin and the CyaA-derived constructs were expressed and purified as described previously (37). In brief, exponential cultures of *E. coli* BL21 λ(DE3) cells were grown at 37 °C in LB medium supplemented with ampicillin (150 μg/ml) to OD_600_ ∼0.6 before induced with 0.5 mM IPTG for additional 4 h of cultivation. The disrupted cells were centrifuged at 4000*g* for 20 min at 4 °C to remove unbroken cells and the supernatant was spun down at 30,000*g* for 30 min at 4 °C. The pelleted inclusion bodies were resuspended in 50 mM Tris-HCl (pH 8.0) and 8 M urea (TU buffer) and the clarified urea extracts after centrifugation at 30,000*g* for 30 min at 4 °C were loaded onto a DEAE-Sepharose column (Sigma, USA) equilibrated with a buffer containing 50 mM Tris-HCl (pH 8.0), 8 M urea, and 120 mM NaCl (TUN). The column was washed with TUN buffer supplemented with 1% Triton X-100 (v/v) followed by extensive washing with the TUN buffer and the LPS-free CyaA proteins were eluted with the TU buffer supplemented with 300 mM NaCl.

The fluorescently labeled CyaA-AC^−^ toxoid was prepared as described previously (27).

The purity of the proteins was monitored by SDS–polyacrylamide gel electrophoresis (SDS-PAGE) and protein concentrations were determined by Bradford assay (Bio-Rad, USA) using bovine serum albumin as a standard.

### Circular dichroism (CD) spectroscopy

The far-UV CD spectra were recorded at 25 °C on a Chirascan-plus spectrometer (Applied Photophysics, USA) in rectangular quartz Suprasil cells of 1-mm path length (110-QS, Hellma, Germany). The Ca^2+^-free protein samples (0.2 mg/ml) were diluted in 20 mM Tris-HCl (pH 8.0) and 50 mM NaCl in the absence or presence of 10 mM CaCl_2_ and measured for wavelengths from 200 to 260 nm with a scanning speed of 1 nm/s. The Ca^2+^-induced protein folding was monitored by stepwise titration of the protein samples (0.2 mg/ml) with increasing concentrations of CaCl_2_ (0–1.5 mM). Spectra of the buffers were subtracted from the protein spectra and the residual weight ellipticity (Θ) was expressed in degrees square centimeter per decimole [deg.cm^2^.dmol^−1^].

### Thermal stability

Thermal stability assays were performed by nanodifferential scanning fluorimetry (nanoDSF) using a Prometheus NT.48 instrument (NanoTemper Technologies, Germany). The Ca^2+^-loaded protein samples (0.2 mg/ml) were diluted in 5 mM Tris-HCl (pH 8.0), 50 mM NaCl, and 1.5 mM CaCl_2_ and loaded into nanoDSF grade standard capillaries (NanoTemper Technologies, Germany). The measurements were conducted from 20 to 95 °C (with a temperature ramp of 2 °C/min) under constant monitoring of tryptophan fluorescence at 350 and 330 nm. The melting temperature (*Tm*) values, corresponding to the inflection points of the unfolding curve, were determined by using a PR.ThermControl (NanoTemper Technologies, Germany).

### Small angle X-ray scattering (SAXS)

The SAXS data were collected using a PILATUS 2 M pixel detector (DECTRIS) at the P12 beamline of the EMBL (DESY) at a sample–detector distance of 3.1 m and a wavelength of 1.24 Å (39). With this setup, arrangement of momentum transfer of 0.0028< s< 0.45 Å^−1^ is covered (s=4πsin(θ)/λ, where 2θ is the scattering angle). Protein samples were measured at 10 °C in 10 mM Tris-HCl (pH 8.0), 150 mM NaCl, 10 mM CaCl_2_ at four different concentrations ranging from 1 to 8 mg/ml and from 0.625 to 5 mg/ml for RTX-1 and RTX-2, respectively. The scattering data were acquired as 20 successive 50 ms frames (with total exposure of 1 s). No radiation damage was observed. The scattering from the buffer was collected before and after each protein sample and the average of the data was normalized and background substracted using PRIMUS (40). The low-angle data collected at lower concentrations were extrapolated to infinite dilution and merged with the higher concentration data to yield the final composite scattering curve. Forward scattering intensity (I_0_) and radius of gyration (R_*g*_) were calculated using the Guinier approximation, considering that for a very small range of momentum transfer values (s < 1.3/R_*g*_), the intensity is represented as I_s_ =I_0_ exp(-s^2^R_*g*_^2^/3). The pair-distance distribution function *p*_r_ was computed using GNOM (41) and used to estimate the maximum particle dimension (D_max_). Molecular mass of proteins was estimated from I_0_ (calculated from Guinier approximation) using a reference solution of bovine serum albumin (66 kDa). Low-resolution *ab initio* models were calculated using DAMMIF (42) that employs simulated annealing for determination of the shape of the macromolecule represented as densely packed beads within a sphere with a diameter (D_max_) minimizing the discrepancy χ^2^ between experimental scattering curve and the one corresponding to the model. For each construct 20 initial models were calculated, clustered, and averaged with DAMCLUST (43).

### Competition assay in CHO-CR3 cells

Competitive binding of the proteins in the presence of the Dy647-labeled CyaA-AC^−^ was determined by flow cytometry as previously described (30). In brief, aliquots of CHO-CR3 cells (1 × 10^5^) were incubated on ice for 15 min in a final volume of 0.2 ml of HEPES-buffered salt solution (10 mM HEPES, pH 7.4, 140 mM NaCl, 5 mM KCl) complemented with 2 mM CaCl_2_, 2 mM MgCl_2_, 1% (w/v) glucose, and 1% (v/v) FCS in the presence of the tested competitor proteins. A range of 0–175 nM of each protein was assessed. Afterward, the cells were incubated with Dy647-labeled CyaA-AC^−^ (5.6 nM) for 30 min on ice and analyzed by flow cytometry (FACS LSR II, BD Biosciences, USA) in the presence of 1 μg/ml of Hoechst 33258 vital dye. Data were analyzed using the FlowJo software (Tree Star, USA) and appropriate gating was used to exclude cell aggregates and dead cells. Binding data were deduced from the mean fluorescence intensities (MFIs) of the cell-associated Dy647-labeled CyaA-AC^−^, where MFI of the cell-bound Dy647-labeled CyaA-AC^−^ in the absence of competitor protein was taken as 100%.

### CyaA binding

The CHO-CR3 or THP-1 cells (10^6^/ml) were incubated in D-MEM with CyaA proteins (5 nM) at 4 °C for 30 min, washed extensively to remove unbound proteins, and lyzed with 0.1% Triton X-100. The cell-associated AC enzyme activity was measured in the presence of 1 μM calmodulin as previously described (44). One unit of AC activity corresponds to 1 μmol of cAMP per min at 30 °C (pH 8.0).

### Determination of intracellular cAMP levels

Twofold dilutions of CyaA proteins were exposed to aliquots of CHO-CR3 (10^5^/ml) or THP-1 cells (1.5 × 10^5^/ml) in D-MEM medium at 37 °C for 30 min before the reaction was stopped by addition of 0.2% Tween-20 in 100 mM HCl. The samples were heated at 100 °C for 15 min, neutralized by 150 mM unbuffered imidazole, and cAMP levels were determined by a competitive immunoassay as described previously (45).

### CyaA activity on sheep erythrocytes

Cell binding, invasive and hemolytic activities of CyaA proteins on sheep erythrocytes (LabMediaServis, Czech Republic) were determined as described previously (7, 46). In brief, binding of CyaA to sheep erythrocytes was determined by quantitating the amount of cell-associated AC activity (membrane-bound CyaA). Cell-invasive activity of CyaA was measured by determining the AC activity protected against externally added trypsin upon internalization into erythrocytes. Hemolytic activity was determined spectrophotometrically at 541 nm (A_541 nm_) as the release of hemoglobin after incubation of sheep erythrocytes (5 × 10^8^/ml) with 50 nM CyaA proteins at 37 °C for 4 h. The activity of intact CyaA was taken as 100%.

### Mass spectrometry (MS)

The acylation status of the proteins was determined as described previously (6). Briefly, the proteins were digested in 25 mM ammonium bicarbonate (pH 8.2) and 4 M urea at a trypsin/protein ratio of 1:50 for 6 h at 30 °C followed by addition of the second portion of trypsin to a final ratio of 1:25 and incubation for another 6 h at 30 °C. The MS analyses were performed on a SolariX XR FT-MS instrument equipped with a 15 T superconducting magnet and a Dual II ESI/MALDI ion source (Bruker Daltonics, Germany) operating in survey LC-MS mode. The instrument was calibrated online using Agilent tuning mix, resulting in mass accuracy below 2 ppm. MS spectra were processed using the DataAnalysis 4.4 software package (Bruker Daltonics, USA) and the extracted data were searched against a single corresponding toxin molecule using the Linx software (RRID:SCR_018657). The acylation status of the K860 and K983 residues was determined from relative intensities of acylated peptide ions and their unmodified counterparts.

### Animal experiments

All animal experiments were approved by the Animal Welfare Committee of the Institute of Molecular Genetics of the Czech Academy of Sciences, v. v. i., in Prague, Czech Republic. Handling of animals was performed according to the *Guidelines for the Care and Use of Laboratory Animals*, the Act of the Czech National Assembly, Collection of Laws no. 246/1992. Permission no. 19/2020 was issued by the Animal Welfare Committee of the Institute of Molecular Genetics of the Czech Academy of Sciences in Prague.

Five-week-old female BALB/cByJ mice (Charles River, France) were immunized by intraperitoneal injection with either the Ca^2+^-free or Ca^2+^-loaded proteins (1.5 μM in 200 μl) adjuvanted with aluminum hydroxide (Alum, SevaPharma, Czech Republic) and incomplete Freund′s adjuvant (Sigma, USA), respectively. Control mice were vaccinated with the adjuvants with PBS. Mice received two doses of the vaccines in 2 weeks interval and 2 weeks after the second immunization, the blood was collected from anesthetized animals (i.p. injection of 80 mg/kg ketamine and 8 mg/kg xylazine) by retroorbital puncture method. Sera were recovered from the supernatant after centrifugation of clogged blood at 5000*g* for 10 min at 8 °C and stored at –20 °C.

## Data availability

The SAXS data and models of the RTX-1 and RTX-2 constructs are deposited in the Small-Angle Scattering Biological Data Bank (http://www.sasbdb.org) under the accession code SASDL62 and SASDL72, respectively.

## Conflict of interest

The authors declare that they have no conflicts of interest with the contents of this article.
